# Multimodal Learning and Intelligent Prediction of Symptom Development in Individual Parkinson’s Patients

**DOI:** 10.3390/s16091498

**Published:** 2016-09-14

**Authors:** Andrzej W. Przybyszewski, Mark Kon, Stanislaw Szlufik, Artur Szymanski, Piotr Habela, Dariusz M. Koziorowski

**Affiliations:** 1 Polish-Japanese Academy of Information Technology, 02-008 Warszawa, Poland; artur.szymanski@pjwstk.edu.pl (A.S.); piotr.habela@pjwstk.edu.pl (P.H.); 2 Department Neurology, University of Massachusetts Medical School, Worcester, MA 01655, USA; 3 Mathematics and Statistics, Boston University, Boston, MA 02215, USA; mkon@bu.edu; 4 Neurology, Faculty of Health Science, Medical University of Warsaw, Warszawa 03-242, Poland; stanislaw.szlufik@gmail.com (S.S.); dkoziorowski@esculap.pl (D.M.K.)

**Keywords:** neurodegenerative disease, rough set, machine learning, decision rules

## Abstract

We still do not know how the brain and its computations are affected by nerve cell deaths and their compensatory learning processes, as these develop in neurodegenerative diseases (ND). Compensatory learning processes are ND symptoms usually observed at a point when the disease has already affected large parts of the brain. We can register symptoms of ND such as motor and/or mental disorders (dementias) and even provide symptomatic relief, though the structural effects of these are in most cases not yet understood. It is very important to obtain early diagnosis, which can provide several years in which we can monitor and partly compensate for the disease’s symptoms, with the help of various therapies. In the case of Parkinson’s disease (PD), in addition to classical neurological tests, measurements of eye movements are diagnostic. We have performed measurements of latency, amplitude, and duration in reflexive saccades (RS) of PD patients. We have compared the results of our measurement-based diagnoses with standard neurological ones. The purpose of our work was to classify how condition attributes predict the neurologist’s diagnosis. For *n* = 10 patients, the patient age and parameters based on RS gave a global accuracy in predictions of neurological symptoms in individual patients of about 80%. Further, by adding three attributes partly related to patient ‘well-being’ scores, our prediction accuracies increased to 90%. Our predictive algorithms use rough set theory, which we have compared with other classifiers such as Naïve Bayes, Decision Trees/Tables, and Random Forests (implemented in KNIME/WEKA). We have demonstrated that RS are powerful biomarkers for assessment of symptom progression in PD.

## 1. Introduction

Experienced neurologists use their knowledge together with statistical intuition to study symptom development in Parkinson’s disease (PD). By applying statistics to large databases, one can find significant information about the specificities of PD. However, due to the great variety of patients symptoms and treatments, some results obtained even from the most prominent experts can be inconsistent. Applying the most popular statistical averaging methods to such inconsistent conclusions may give confusing results, even leading to statements that specific care regimens do not effectively influence “average” PD patients. We may face similar problems when explaining factors resulting in longer, better, and more active lives for people with Parkinson’s. Neurologists from different centers may also differently interpret the meanings of Unified Parkinson’s Disease Rating Scale (UPDRS) scores, leading to differing therapies. These problems are expressed in the popular statement “No two people face Parkinson’s in quite the same way”. People diverge substantially in their combinations of symptoms, rates of progression, and reactions to treatment. As mentioned above, averaging patients’ symptoms as effects of different therapies gives very crude approximations of results. If we want to improve such investigations, we need to take into account an excessive diversity of patients’ symptoms and the changeable effects of cares and therapies in distinctive PD cases. Here we follow an earlier proposal [1] to extend statistical analysis by data mining and machine learning (ML) methods, which can give a higher meaning to an individual patient’s symptoms and singular PD development. As a consequence, our methods will suggest a specific treatment adjusted to different individual patients that may lead in slowing down symptom progression and improvement of quality of life. Proposed treatments’ improvements will be based on the granularity of the individual patient’s data (as explained in the Methods section).

Our symptom classification method can be compared with complex object recognition in the visual system. The latter’s ability to recognize various objects corresponds roughly to the afferent ascending pathways that classify object parts at simple levels in lower areas, and more complex ones in higher areas. In many implementations of such systems, the logic is assumed to be based on ‘and’ conjunctions where several components are necessary for a given classification. These hierarchical classifications are integrated through analysis of whole object (“holistic”) visual properties at all levels by descending pathway influences [2], and that follow a complementary ‘or’ logic. In our symptom classification system, we use rough set (RS) theory, an approach related to granular computing in which a granule contains all attributes (as in ‘and’ logic) and where interactions between granules are related to ‘or’ logic [2]. These interactions at multiple levels between measurement and knowledge will be used to differentiate individual symptoms and treatment variability in a way that parallels in the above models the process of complex object inspection [3,4]. With the use of machine learning algorithms, we will determine whether sometimes subtle symptom variations are significant enough to assist in improving individual patients’ treatments.

Diagnostic findings and neurological test interpretations of individual Parkinson’s patients depend to some extent on neurologists’ experience. In most cases, they estimate values of the Hoehn, Yahr, and UPDRS scales, not very precisely and in partly subjective ways. In our data mining-based system, we have used neurologists’ diagnoses as decision attributes and measurements as condition attributes.

## 2. Materials and Methods

Our experiments were performed on 10 PD patients who had undergone Deep Brain Stimulation (DBS) surgery mainly related to their On-Off sensitivity to a medication (L-Dopa). They were qualified for the surgery and observed postoperatively in the Dept. of Neurology, Medical University of Warsaw and received surgical DBS in the Institute of Neurology and Psychiatry at University of Warsaw (WUM) [5]. We obtained horizontal reflexive saccade (RS) measurements in PD patients during four sessions designed as S1: MedOffDBSOff, S2: MedOffDBSOn, S3: MedOnDBSOff, S4: MedOnDBSOn. During the first session (S1), the patient was off medication (L-Dopa) and the DBS stimulator was Off; in the second session (S2), the patient was off medication, but the stimulator was ON; in the third session (S3), the patient had completed his/her doses of L-Dopa and the stimulator was Off, and in the fourth session (S4), the patient was on medication with the stimulator ON. Changes in motor performance, behavioral dysfunction, cognitive impairment, and functional disability were evaluated in each session according to the UPDRS. The reflexive saccades (RS) were recorded by a head-mounted saccadometer (Ober Consulting, Poland) that uses an infrared eye tracking system coupled with a head tracking system (JAZZ-pursuit—Ober Consulting, Poland). This has a sampling frequency of 1000 Hz, thus yielding high accuracy and precision in eye tracking. Thanks to a head-mounted sensor, it is possible to compensate for possible head movements relative to the monitor.

A patient was sited at a distance of 60–70 cm from the monitor with head supported by a headrest in order to minimize head motion. We measured fast eye movements in response to a light spot switched on and off, moving horizontally from the straight eye fixation position (0°) to 10° to the left or 10° to the right after arbitrary times ranging between 0.5–1.5 s. When the patient fixated eyes on the spot in the middle marker (0°) the spot then changed color from white to green, indicating a signal for performance of RS reflexive saccades (RS); or from white to red meaning a signal for performing antisaccades (AS). Then the central spot was switched off and one of the two peripheral targets, selected at random with equal probability, was illuminated instead. There was no time lag between switching off of the central spot and switching on the peripheral target (gap = 0 task). Patients had to look at the targets and follow them as they moved in the RS task or made opposite direction saccades in the AS task. After making a saccade to the peripheral target, the target remained on for 0.1 s, after which another trial was initiated.

In each test, the subject had to perform 20 RS and 20 AS in a row in MedOff (medication off), within two situations: with DBSOff (S1) and DBSOn (S2). In the next step, the patient took medication and had a break for one-half to one hour, and then the same experiments were performed, with DBSOff (S3) and DBSOn (S4). In this work, we have analyzed only RS data using the following population parameters averaged for both eyes: delay mean (±SD) (standard deviation); amplitude mean (±SD); max velocity mean (±SD); duration mean (±SD).

### 2.1. Theoretical Basis

The structure of data is an important point of our analysis. Here, we represent them in the form of an information system or a decision table. We define such an information system [6] as a pair S = (U, A), where U, A are nonempty finite sets called the universe of objects and the set of attributes, respectively. If a∈A and u∈U, the value a(u) is a unique element of V (where V is denoted as the value set).

The indiscernibility relation of any subset B of A or IND(B), is defined [6] as follows: (x,y)∈ IND(B) or xI(B)y if and only if a(x)=a(y) for every a∈B, where a(x)∈V. IND(B) is an equivalence relation, and [u]_B_ is the equivalence class of u, or a B-elementary granule. The family of all equivalence classes of IND(B) will be denoted U/I(B) or U/B. The block of the partition U/B containing u will be denoted by B(u).

We define a **lower approximation** of symptoms set X⊆U in relation to a symptom attribute B as $BX=\left\{u\in U:{\left[u\right]}_{B}\subseteq X\right\}$, and the **upper approximation** of X as $\bar{B}X=\left\{u\in U:{\left[u\right]}_{B}\cap X\neq 0\right\}$. In other words, all symptoms are classified into two categories (sets). The lower movement approximation set X has the property that all symptoms with certain attributes are part of X, and the upper movement approximation set has property that only some symptoms with attributes in B are part of X (for more details see [7]). The difference between the uppper and lower approximations of X is defined as the boundary region of X: BN_B_ (X). If BN_B_ (X) is empty then X is exact (crisp) with respect to B; otherwise if BN_B_ (X) ≠ϕ then X is not exact (i.e., is rough) with respect to B. We say that the B-lower approximation of a given set X is union of all B-granules that are included in X, and the B-upper approximation of X is of the union of all B-granules that have nonempty intersection with X.

The system S will be called a decision table S = (U, C, D) where C is the condition and D is the decision attribute [6,7]. In the decision table, the decision attribute D is placed in the last column, and condition attributes measured by the neurologist, are placed in other columns. On the basis of each row in the table, rules describing the condition of each patient can be proposed. As the number of rules is the same as the number of rows, these rules can have many particular conditions. The main concept of our approach is to describe different symptoms in different patients by using such rules.

However, as there are large inconsistencies between disease progression and symptoms between individual patients, and effects of similar treatments are not always the same. In order to describe such differences **our rules must have some “flexibility”, or granularity**. In other words, one can also think about the probability of finding certain symptoms in a certain group of patients. **This approach, called granular computation, should simulate the way in which experienced neurologists might interact with patients.** It is the ability to perceive a patient’s symptoms at various levels of granularity (i.e., abstraction) in order to abstract and consider only those symptoms that are universally significant and serve to determine a specific treatment(s). Looking into different levels of granularity determines the different levels of knowledge abstraction (generality), as well as assisting with greater understanding of the inherent knowledge structure. Granular computing simulates human intelligentce related to classification of objects in the sensory system (e.g., vision [2,3,4,5]) and motor [1] system as well as in task-specific problem solving behaviors.

We have also defined the notion of a reduct and template [1] as a basis for the decomposition. A decomposition tree is defined as a binary tree, whose every internal node is labeled by some template and external node (leaf) is associated with a set of objects matching all templates in a path from the root to a given leaf [8].

In our tests, we have divided our data into two or more subsets (mostly to four or six subgroups —four- or six-folding cross-validation tests). By training on all except one of these subgroups (the training set) using machine learning (ML), we have obtained classifiers that when applied to the remaining (test) set gave new numerical decision attributes. In the next step, we have correlated decision attributes obteined by ML with neurologist decision attributes in order to get a confusion matrix.

### 2.2. Computational Basis

We have extended our previous work [1] with new classification methods that were performed with the help of the KNIME Analytics Platform and WEKA. As a result, we have added new algorithms including:

- Random Forest (two implementations: KNIME and WEKA) [9]
- Decision Tables (two implementations: KNIME and WEKA) [10]
- Bayes classifier (KNIME implementation) [11]
- Tree ensembles (KNIME implementation)

Tree ensemble models build classifiers based on ensembles of the decision trees that may be seen as random forest variants. As in the random forest model, training can be done on different sets of rows (records) and/or columns (describing attributes). The output model describes an ensemble of decision trees and is implemented by a simple majority vote.

We have applied certain sets of parameters for each of the above-mentioned algorithms. For the naïve Bayes classifier, we have use 0 as the default probability and 20 as the maximum number of nominal values per attribute. For the decision tree, we have used the gini index as a quality measure, no pruning with the reduced error option, a minimum number of records was 2, a maximum number of records per view was 1000, an average split point and the skipping of nominal columns without domain information. In the case of the WEKA implementation, we used the following evaluation metrics: accuracy (for discrete classes) and root mean square error (for numeric classes). For tree ensembles and the random forest (where applicable), we used the information gain ratio as split criteria, mid plot split, no tree limit, minimum split size and child node size, square root attribute sampling for columns, 100 of model number, different attribute set for each tree node. In the case of WEKA implementation, the number of trees was limited to 10.

Input data for those algorithms were prepared using native KNIME nodes. Processing included as it was in the original work discretization function. It is important to note that the discretization function in KNIME is much simpler that the one used in RSES. In comparison, the more sophisticated RSES discretizes functions, generating cuts based on decision attributes [12]; it bins numerical values into groups with equal frequencies.

In addition to the standard accuracy coefficient, we have also calculated from the confusion matrix the Matthews correlation coefficient that is a correlation coefficient between the observed and predicted binary classifications. We also have used SMOTE in order to oversample our data, and we have used nearest neighbor 3 with oversample by three parameters.

### 2.3. Dedicated Database

In the course of this study, a dedicated database for patients and measurement data has been designed and implemented. Given the number of patients under consideration and a moderate volume of the processed data, this project did not pose significant data management challenges. However, both the immediate needs of data analysis, and plans for the future maintenance of the dataset and the development of the related data collection and retrieval functionality, brought some important architectural and functional requirements.

To help protect the data integrity, both the alphanumerical data as well as the associated files (in the form of Binary Data Objects) are kept under the control of the database management system. The core structure of the database is designed to match the exact needs of the current research. It consists of the anonymized patient data, their associated history of examinations, and finally their measurement variants—with respect to the DBS (deep brain stimulation) and BMT (medication) on/off configuration. Given the number of the attributes and the evolution of their set in the early phase of the project, it became necessary to redesign the data structure to allow a greater degree of generality. Hence the structure is open for further extensions in terms of:

- Defining new attributes describing the Patient, Examination, and Measurement entities;
- Defining associated constraints or the dictionary of enumerative values for those attributes;
- Defining new types of files for storing additional information regarding Patients, Examinations, and Measurements;
- Establishing a numbering and naming convention for the instances of the above mentioned entities and files.

Although the current setup involves only the data collection and interface by a single team, the software is being prepared for functioning in a more distributed and versatile manner. The functionality under development includes:

- Web service interfaces for contributing measurements made with mobile devices;
- Advanced privilege management functionality to allow multiple-team collaboration and support for different access levels;
- Internationalization in terms of user interface language and data and metadata language;
- Integration with the human motion database, to extend the scope of future examination.

The database is currently operated by PJAIT and is to be maintained after the completion of the current project.

## 3. Results

### 3.1. Neurological Tests and Eye Movement Measurements

The mean age of the patients was 51.1 ± 10.2 (SD) years, mean disease duration was 11.3 ± 3.2 years, mean UPDRS (related to all symptoms) were: S1: 66.6 ± 13.8; S2: 30.0 ± 16.3; S3: 58.1± 13.5; S4: 22.3 ± 13.6; mean UPDRS III (related only to motor symptoms): S1: 42.7 ± 11.3; S2: 17.8 ± 10.6; S3: 34.1 ± 10.8; S4: 10.9 ± 8.3; mean RS latencies: S1: 291.2 ± 93.1 ms, S2: 199.6 ± 39.5 ms, S3: 232.9 ± 82.7 ms; S4: 183.2 ± 30 ms.

**Figure 1:**
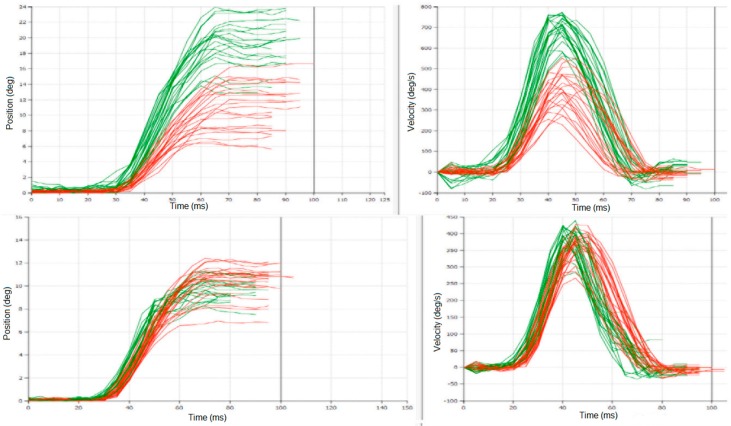
An example of experimental recordings from Pat #38 in two sessions: upper plots from session S1: MedOFF & StimOFF; lower plots from session S4: MedON & StimON; left plots show latency measurements, right plots—saccade amplitude and velocity. Notice change in variability of responses between S1 and S4. These plots were obtained directly from the saccadometer (Ober Consulting), different colours are related to different eyes.

Differences between latencies: S1-S2, and S1-S4 were statistically significant (*t*-test *p* < 0.01) even when they were variable in individual patients (Figure 1); meanwhile S1-S3 was not statistically significant; this is similar to differences between UPDRS/UPDRS III: S1-S2 and S1-S4 were statistically significant (*p* < 0.001), and S1-S3 was not statistically significant.

Other parameters of RS did not change significantly with the session number.

### 3.2. Rough Set and Machine Learning Approach

As described above, we have used the RSES 2.2 (Rough System Exploration Program) [8] in order to find regularities in our data. At first, our data were placed in an information table (Table 1) as originally proposed by Pawlak [6].

The full table has 15 attributes and 36 objects (measurements). In Table 1 are values of 11 attributes for two patients: P#—patient number, age—patient’s age, sex—patient’s sex: 0—female, 1—male, t_dur—duration of the disease, S#—Session number, UPDRS—total UPDRS, HYsc—Hoehn and Yahr scale all measured by the neurologist and saccades measurements: SccDur—saccade duration; SccLat—sac-cade latency; SccAmp—saccade amplitude, and SccVel—saccade velocity.

In the next step, we have performed reduction of attributes (see reduct in the Methods section) to a minimum number of attributes describing our results. We have also created a discretization table: here, single values of measurements were replaced by their range (as describe in the Method section on cut sets). As the result we have obtained the decision table (Table 2—see below).

In the first column is the patient number, in the second the patient age, dividing our group into patients below (Pat#28) or above (Pat#38) 55 years of age; disease duration and the Hoehn and Yahr scale were not considered important (stars), along with session number. Other parameters of saccades were also divided into ranges. It is interesting to note how the UPDRS were divided into different ranges: above 55, 22.5 to 55, 14 to 22.5, and below 14 (the last column). On the basis of this decision table, we can write the following rule:

(‘Pat’ = 38) & (‘age’ = “(55.0, Inf)”) & (‘Sess’ = 4) & (‘SccDur’ = “(−Inf, 45.5)”) & (‘SccLat’ = ”(−Inf, 260.0)") & (‘SccAmp’) = “(−Inf ,10.5)”) => (‘UPDRS’ = “(−Inf, 14.0)”)
(1)

We read this formula above (Equation (1)), as stating that each row of the table (Table 2) can be written in form of such equation. It states that if we evaluate patient #38 and with age above 55 years and in session #4 and with saccade duration below 45.5 ms and saccade latency below 260 ms and … and saccade amplitude below 10.5, then the patient’s UPDRS is below 14.

#### 3.2.1. Estimation of UPDRS on the Basis of Eye Movement Measurements

In the next step, we need to find common rules for these equations by using a data mining system based on rough set theory [6]. We have tested our rule using machine-learning (ML) algorithms. At first, we have randomly divided our data into six groups (so-called a six-fold cross-validation method). We took five groups as a training set and tested the sixth one. By changing groups belonging to the training and test sets, we have removed the effect of accidental group divisions. The results of each test were averaged. The results are given as a confusion matrix (Table 3). In this case, as a ML algorithm that gave the best results we have used the decomposition tree (see the Methods section).

#### 3.2.2. Estimation of UPDRS on the Basis of Eye Movement and Additional Measurements

Next, we extended our tests (in comparison with [1]) by adding to our dataset additional attributes, including:

- PDQ39—quality of life measure
- AIMS—Abnormal Involuntary Movement Score
- Epworth—sleep scale

A significant increase in accuracy was achieved in prediction of Total UPDRS by adding the PDQ attribute. We were able to achieve 90.3% of accuracy with 47% coverage. We can see confusion matrix for this classification in Table 4.

**Figure 2:**
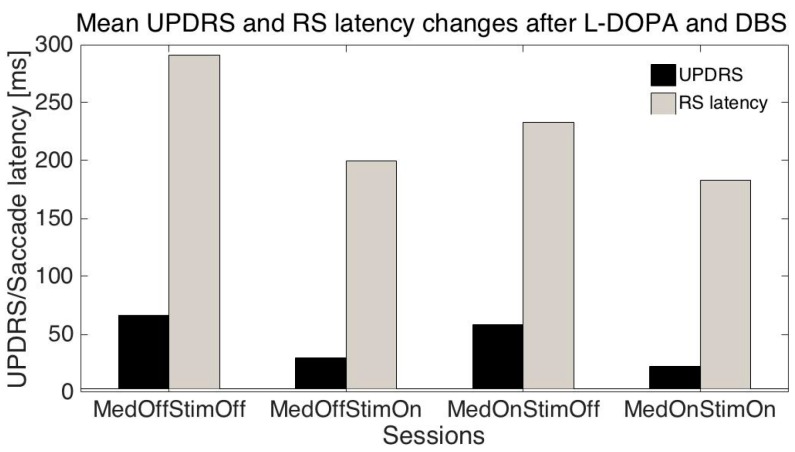
This graph shows parallel changes in UPDRS and reflexive saccade latencies as functions of medication and stimulation. Changes between control and MedOnStimOn were significantly different for UPDRS *p* < 0.001, RS *p* < 0.01.

We have performed several tests trying to predict UPDRS values on the basis of measured saccade properties. As changes in UPDRS and saccade latencies were similar when the session number was changed (Figure 2) we tried to predict individual UPDRS values only from RS latencies. When the session number, patient age, RS latency, amplitude, and duration were added, the global accuracy in UPRDS prediction was about 80% (ML: decomposition tree, cross-validation-method). As UPDRS is a standard measurement in PD, the above results give the possibility of at least partly replacing or augmenting neurologist estimates with the eye movements (EM) measurement results.

#### 3.2.3. Estimation of Different Therapies Effects

Another question that arises is whether EM can help to estimate possible effects of different treatments in individual patients. In order to demonstrate an answer, we have removed EM measurements and added other typically measured attributes such as: Schwab and England ADL Scale, and UPDRS III and UPDRS IV to the decision table and tried to predict the effects of different treatments as represented by sessions 1 to 4 (medication and stimulation effects). Table 5 is a part of full decision discretized-table with decision attributes—the session number placed in the last column. On the basis of this table, we have formulated rules using a rough set system and tested them with randomly divided data into six groups. We took five as a training set (using ML protocol) and tested with sixth. In order to remove effect of the accidental division, we have exchanged training and test groups and averaged results placed in the confusion matrix (Table 6).

TPR: True positive rates for decision classes, ACC: Accuracy for decision classes: the global coverage was 0.64, **the global accuracy was 0.53**, coverage for decision classes: 0.5, 0.5, 0.75, 0.7.

We have performed the same procedures once more (results of discretization are in Table 7) in order to test results of patients’ eye movement influence on our predictions (presented as a confusion matrix in Table 8).

TPR: True positive rates for decision classes, ACC: Accuracy for decision classes, the global coverage was 0.5; **the global accuracy was 0.91**; coverage for decision classes: 0.6, 0.6, 0.6, 0.25.

As above, results of each test were averaged in a six-fold cross-validation resulting a confusion matrix (Table 8). As a machine-learning algorithm, we have used the decomposition tree (see Methods). In the next step, we have extended our data set by three additional parameters: PDQ39, AIMS, Epworth and performed discretization as is shown below in Table 9.

By extending our dataset with additional attributes we were able to achieve 4% more in accuracy for session prediction that make it **94.4%** level. The confusion matrix for this test can be found in Table 10.

#### 3.2.4. Estimation of UPDRS II and UPDRS III on the Basis of Eye Movement and Additional Measurements

Further on, we have extended our original work [1] by creating models for predicting other attributes; the best results we were able to achieve for UPDRS II and III, reaching 85% accuracy and 67.7% coverage for UPDRS II, and 81.7% accuracy and 61.1% coverage for III. Below we can see detailed discretized tables used in those models (Table 11 for UPDRS III) as well as confusion matrixes (Table 12 for UPDRS III, Table 13 for UPDRS II).

When we have compared above results for predicting accuracy for UPDRS II to predictions without PDQ, AIMS, and Epworth attributes, and we have noticed decreased accuracy in predictions from 78.7% to 62.2% (16.4% difference).

### 3.3. Comparison of Rough Set Based and Other Classifiers

Our ultimate purpose was to compare our models built using RS methods with other well-known algorithms. To accomplish this, we have used the same information tables and processed them with different classification algorithms as described in the methods section and results are shown below in Table 14.

In summary, our new results have demonstrated that adding eye movement (EM) measurements to classical neurological data performed as the ‘gold standard’ by the most clinicians, improved predictions of disease progression, as measured by improvement in global accuracy from 0.53 to 0.91. The EM measurements may also partly replace some standard neurological measurements such as the UPDRS, as global accuracy of the total UPDRS predictions taken from EM data was 0.79 for our 10 PD patients. We have also performed comparisons to other well-known ML algorithms showing competitiveness of the RS algorithm (Table 14). All data from this study are shown in Table A1 (Appendix A). We have also tested influence of different training and testing sets on the accuracy of our classifications (Table B1, Table C1 in Appendix B, Appendix C). We have used four-, five-, six-fold, and leave-one-out (40-fold for our dataset) tests. In the majority of tests for different classifiers (Table B1 in Appendix B), the six-fold gave similar accuracy to four- or five-fold and leave-one-out tests. However, for the rough set based classification in the majority of different parameters tests six-fold trials were the best.

As our database is relatively small, we did not perform direct subsampling only oversampling by 3 with help of SMOTE. It was surprising that oversampling gave the best results for all algorithms. It is probably related to the fact that our dataset is small, as our inter-rater quantitative agreement measured by the Cohen’s kappa was substantial (0.7) for all test with WEKA-Decision Table.

We have also calculated the Matthews correlation coefficient (MCC) as another measure of prediction accuracy, which can be interpreted as a correlation coefficient between the observed and predicted classifications (Table B1 and Table C1). We have got significant MCC with rough set based classification for UPDRS and session number (0.85 and 0.95—Table C1 in Appendix C), but for other algorithms only for the oversampled data (Table B1 in Appendix B).

## 4. Discussion

As mentioned above, there are significant differences between symptom developments and effects of different treatments in individual PD patients. As a consequence, even with large numbers of approaches and clinical trials, there still have been few conclusive results on direct therapeutic connection to quantitative changes in PD symptoms. There are numerous reasons for such disappointments: first, the limitations of current disease models in target validation and testing; second, difficulties in choosing clinical endpoints; third, problems in finding sensitive biomarkers of disease progression. The major drawback is that disease starts long before an individual patient notices the first disturbing motor symptoms. As mentioned above, a consequence of brain plasticity is compensation for loss of neurons, to an extent that subjects do not realize that problems are occurring in their brains over one or two decades at a time. Another aspect is that, because individual brains develop different specific compensatory mechanisms, individual pathologies form a large spectrum. Therefore, one needs new, more sensitive, possibly automatic and intelligent methods that may help in early diagnosis. In this study, we have given an example comparing classical neurological diagnostic protocols with a new approach. The main difference between these types of measures is in their precision and objectivity. Our approach is doctor-independent, and can be performed automatically and more often than standard doctor’s visits. In the near future, it may help in transforming some hospital-based to home-based treatments. In this scenario, it will be possible to measure patient symptoms at home, and send these for consultation by neurologists. Such methods will be faster, more precise, and can help with more frequent measurements. As a consequence, they may help not only to determine more objectively a patient’s symptoms, but also to follow up disease progression in more frequent intervals, something not possible currently, with the limited time resources of neurologists. If we obtain such information, it may lead to more appropriate therapies and slowing down of disease progression. It is one of the purposes of this work to try to extract knowledge from symptoms in order to further develop more appropriate therapies and better control disease development.

## 5. Conclusions

We have presented a comparison of classical statistical averaging methods related to doctors’ intuition and their PD diagnosis with different AI approaches. We have used processed neurological data from PD patients in four different treatments and have plotted averaged effects of medication and brain stimulation in individual patients. As these effects are strongly patient-dependent, they could not give enough information to predict new patients’ behavior. The AI approaches (such as machine learning and data mining) are more universal, giving general rules for predicting individual patient responses to treatments as demonstrated above (in UPDRS, UPDRS II, UPDRS III, or session number predictions).

## Figures and Tables

**Table 1:** Extract from the information table.

P#	Age	Sex	t_dur	S#	UPDRS	HYsc	SccDur	SccLat	SccAmp	SccVel
28	54	1	8	1	58	2.0	43	402	12	566.9
28	54	1	8	2	40	1.0	46	297	11	474.5
28	54	1	8	2	40	1.0	49	227	10	431.2
28	54	1	8	4	16	1.0	47	198	9	376.2
38	56	0	11	1	49	2.5	42	285	14	675.2
38	56	0	11	2	22	1.5	48	217	12	509.7
38	56	0	11	3	37	2.5	43	380	14	638.9
38	56	0	11	4	12	1.5	45	187	10	482.6

**Table 2:** Part of the decision discretized-table.

P#	Age	t_dur	S#	HYsc	SccDur	SccLat	SccAmp	UPDRS
28	(−Inf, 55.0)	*	1	*	(−Inf, 45.5)	(260.0, Inf)	(10.5, Inf)	(55.0, Inf)
28	(−Inf, 55.0)	*	2	*	(45.5, Inf)	(260.0, Inf)	(10.5, Inf)	(22.5, 55.0)
28	(−Inf, 55.0)	*	2	*	(45.5, Inf)	(−Inf, 260.0)	(−Inf, 10.5)	(22.5, 55.0)
28	(−Inf, 55.0)	*	4	*	(45.5, Inf)	(−Inf, 260.0)	(−Inf, 10.5)	(14.0, 22.5)
38	(55.0, Inf)	*	1	*	(−Inf, 45.5)	(260.0, Inf)	(10.5, Inf)	(22.5, 55.0)
38	(55.0, Inf)	*	2	*	(45.5, Inf)	(−Inf, 260.0)	(10.5, Inf)	(14.0, 22.5)
38	(55.0, Inf)	*	3	*	(−Inf, 45.5)	(260.0, Inf)	(10.5, Inf)	(22.5, 55.0)
38	(55.0, Inf)	*	4	*	(−Inf, 45.5)	(−Inf, 260.0)	(−Inf, 10.5)	(−Inf, 14.0)

**Table 3:** Confusion matrix for UPDRS Total binned into four classes. Column headers represent predicted values and row headers represent actual values.

	55.0, Inf	22.5, 55.0	−Inf, 14.0	14.0, 22.51	ACC
**55.0, Inf**	0.3	0.3	0.0	0.0	0.2
**22.5, 55.0**	0.0	1.5	0.0	0.0	1.0
**−Inf, 14.0**	0.0	0.3	0.0	0.2	0.0
**14.0, 22.5**	0.0	0.0	0.0	0.0	0.0
**TPR**	**0.2**	**0.8**	**0.0**	**0.0**	

TPR: True positive rates for decision classes, ACC: Accuracy for decision classes: the global coverage was 0.44, the global accuracy was 0.79, coverage for decision classes: 0.2, 0.5, 0.3, 0.0.

**Table 4:** Confusion matrix for total UPDRS prediction with PDQ, AIMS, and Epworth attributes. Column headers represent predicted values, and row headers represent actual values.

	“(55, 94]”	“(25, 40]”	“[6, 25]”	“(40, 55]”	ACC
**“(55, 94]”**	0.5	0.17	0	0	**0.417**
**“(25, 40]”**	0	0.5	0	0	**0.5**
**“[6, 25]”**	0	0	1.17	0	**1**
**“(40, 55]”**	0	0	0.17	0.33	**0.333**
**TPR**	**0.5**	**0.42**	**0.94**	**0.33**	

**Table 5:** Part of the decision discretized-table **without** eye movement measurements.

Pat#	Age	t_dur	SEngs	UPDRS III	UPDRS IV	UPDRS	Sess#
(27.5, 41.5)	(43.5, Inf)	*	(−Inf, 75)	(36.0, 46.0)	(10.5, Inf)	(1.75, Inf)	1
(27.5, 41.5)	(43.5, Inf)	*	(75, Inf)	(13.0, 26.0)	(10.5, Inf)	(−Inf, 1.75)	2
(27.5, 41.5)	(43.5, Inf)	*	(75, Inf)	(−Inf, 6.0)	(10.5, Inf)	(−Inf, 1.75)	4
(27.5, 41.5)	(43.5, Inf)	*	(−Inf, 75)	(26.0, 36.0)	(10.5, Inf)	(1.75, Inf)	1
(27.5, 41.5)	(43.5, Inf)	*	(75, Inf)	(13.0, 26.0)	(10.5, Inf)	(−Inf, 1.75)	2
(27.5, 41.5)	(43.5, Inf)	*	(−Inf, 75)	(13.0, 26.0)	(10.5, Inf)	(1.75, Inf)	3
(27.5, 41.5)	(43.5, Inf)	*	(75, Inf)	(−Inf, 6.0)	(10.5, Inf)	(−Inf, 1.75)	4

Where * (star) is related to parameter(s) excluded (not significant) in the discretization process.

**Table 6:** Confusion matrix for different session numbers (S1–S4). Column headers represent predicted values and row headers represent actual values.

	1	2	3	4	ACC
1	0.5	0.0	0.5	0.0	0.3
2	0.0	0.5	0.0	0.3	0.4
3	0.8	0.0	0.2	0.0	0.2
4	0.0	0.5	0.0	0.5	0.4
TPR	0.3	0.3	0.2	0.4	

**Table 7:** Part of the decision discretized-table with eye movement measurements.

Pat#	Age	SccVel	UPDRS III	HYsc	SccDur	SccLat	SccAmp	Ses#
(27.5, 34.5)	*	(458.5, 578.0)	(36, Inf)	(1.75, Inf)	(38, Inf)	(308.5, Inf)	*	1
(27.5, 34.5)	*	(458.5, 578)	(11.5, 36)	(−Inf, 1.75)	(38.0, Inf)	(−Inf, 308.5)	*	2
(27.5, 34.5)	*	(341.5, 403)	(−Inf, 11.5)	(−Inf, 1.75)	(38, Inf)	(−Inf, 308.5)	*	4
(34.5, Inf)	*	(665.5, Inf)	(11.5, 36.0)	(1.75, Inf)	(38.0, Inf)	(−Inf, 308.5)	*	1
(34.5, Inf)	*	(458.5, 578)	(11.5, 36)	(−Inf, 1.75)	(38.0, Inf)	(−Inf, 308.5)	*	2
(34.5, Inf)	*	(578.0, 665.5)	(11.5, 36)	(1.75, Inf)	(38.0, Inf)	(308.5, Inf)	*	3
(34.5, Inf)	*	(458.5, 578)	(−Inf, 11.1)	(−Inf, 1.75)	(38, Inf)	(−Inf, 308.5)	*	4

**Table 8:** Confusion matrix for different session numbers (S1–S4). Column headers represent predicted values and row headers represent actual values.

	1	2	3	4	ACC
1	0.8	0.0	0.0	0.0	**0.7**
2	0.0	0.7	0.0	0.0	**0.7**
3	0.2	0.0	0.8	0.0	**0.6**
4	0.0	0.2	0.0	0.3	**0.25**
**TPR**	**0.7**	**0.7**	**0.6**	**0.25**	

**Table 9:** Part of the discretized decision table for session prediction. As we can see for this dataset only PDQ scale give significant value for prediction results.

Pat#	UPDRS_III	SEsc	SccLat	SccDur	SccAmp	PDQ39	Ses#
25	(36.0, 44.5)	(−Inf, 85.0)	(207.64, Inf)	(42.5, Inf)	(9.9, Inf)	(−Inf, 78.5)	1
63	(13.0, 26.0)	(85.0, Inf)	(207.64, Inf)	(42.5, Inf)	(−Inf, 9.9)	(78.5, Inf)	4
25	(36.0, 44.5)	(−Inf, 85.0)	MISSING	MISSING	MISSING	(−Inf, 78.5)	3
25	(−Inf, 6.0)	(85.0, Inf)	(207.64, Inf)	(42.5, Inf)	(9.9, Inf)	(−Inf, 78.5)	4
63	(26.0, 36.0)	(−Inf, 85.0)	(207.64, Inf)	(−Inf, 42.5)	(9.9, Inf)	(78.5, Inf)	1
all the rest	(36.0, 44.5)	(85.0, Inf)	(207.64, Inf)	(42.5, Inf)	(9.9, Inf)	(−Inf, 78.5)	2
all the rest	(44.5, Inf)	(−Inf, 85.0)	(207.64, Inf)	(−Inf, 42.5)	(−Inf, 9.9)	(−Inf, 78.5)	3

**Table 10:** Table below shows the confusion matrix for extension of our previous results in prediction session. Global accuracy was established on 94.4% with 47% coverage. We used six-fold cross validation method with tree decomposition algorithm. Column headers represent predicted values, row headers represent actual values.

	1	2	3	4	ACC
**1**	0.5	0	0	0	**0.333**
**2**	0	0.83	0	0	**0.5**
**3**	0	0	0.33	0	**0.333**
**4**	0	0.17	0	1	**0.75**
**TPR**	**0.33**	**0.42**	**0.33**	**0.83**	

**Table 11:** Fragment of decision table for UPDRS III model.

Pat#	UPDRS II	Ses#	SccLat	PDQ39	UPDRS III
{11, 14, 25, 27, 28, 41, 45, 64}	(14.5, Inf)	{‘S2’}	(259.695, Inf)	(65.5, Inf)	(14, 24]
{11, 14, 25, 27, 28, 41, 45, 64}	(−Inf, 14.5)	{‘S3’}	(−Inf, 259.695)	(65.5, Inf)	(24, 35]
{11, 14, 25, 27, 28, 41, 45, 64}	(−Inf, 14.5)	*	(−Inf, 259.695)	(65.5, Inf)	[2, 14]
38	(14.5, Inf)	{‘S1’}	(259.695, Inf)	(−Inf, 65.5)	(24, 35]
38	(−Inf, 14.5)	{‘S2’}	(−Inf, 259.695)	(−Inf, 65.5)	(14, 24]
38	(14.5, Inf)	{‘S3’}	(259.695, Inf)	(−Inf, 65.5)	(14, 24]
63	(14.5, Inf)	{‘S1’}	(−Inf, 259.695)	(65.5, Inf)	(24, 35]

**Table 12:** Confusion matrix for UPDRS III model. Classification accuracy 81.7%, 61.1% coverage, 40 examination records. Test method: six-fold cross validation.

	“(35, 63]”	“(14, 24]”	“(24, 35]”	“[2, 14]”	ACC
**“(35, 63]”**	1.33	0	0.33	0	**0.667**
**“(14, 24]”**	0	0	0	0.17	**0**
**“(24, 35]”**	0	0	0.67	0	**0.667**
**“[2, 14]”**	0	0.33	0	0.83	**0.417**
**TPR**	**0.83**	**0**	**0.58**	**0.5**	

**Table 13:** Confusion matrix for UPDRS II classifier. Accuracy 78.6%, coverage 75.7%. 40 examination records classified with decomposition tree and six-fold cross validation method.

	“(19, 25]”	“[1, 10]”	“(13, 19]”	“(10, 13]”	ACC
**“(19, 25]”**	1.33	0	0	0	**0.833**
**“[1, 10]”**	0	0.83	0	0.33	**0.5**
**“(13, 19]”**	0	0	1	0	**0.5**
**“(10, 13]”**	0	0.5	0.33	0.17	**0.056**
**TPR**	**0.83**	**0.58**	**0.5**	**0.08**	

**Table 14:** As we can for those data sets we were able to achieve the most accurate results for the Rough Set-based approach but in most cases there were algorithms that could give predictions on a similar level, with the exception of classifiers for the session. Classifiers were tested on dataset with PDQ attribute.

Classifier	UPDRS Total	UPDRS II	UPDRS III	Sess#
Bayes	53.17%	77.38%	32.54%	0.00%
Decision Tree	50.79%	7.94%	62.30%	30.16%
Random Forest	67.86%	**79.76%**	60.32%	25.00%
Tree Ensemble	65.48%	70.24%	54.76%	25.00%
WEKA-Random Forest	47.62%	73.02%	49.60%	12.30%
WEKA-Decision Table	62.54%	43.25%	77.38%	35.71%
Rough Set	**90.30%**	78.60%	**81.70%**	**94.40%**

## Appendix A

**Table A1:** Input data for our study.

Pat#	YearOfBirth	UPDRS_II	UPDRS_III	UPDRS_IV	UPDRS_Total	HYscale	SchwabEnglandScale	SccLat	SccDur	SccAmp	SccVel	PDQ39	AIMS	Epworth	Session
11	1955	25	45	3	75	4	60	259.1	51.9	14.5	552.2	43	2	8	1
11	1955	10	17	3	32	1	90	204.4	51.6	14.3	549.7	43	2	8	2
11	1955	25	32	3	62	4	60	314.6	46.2	12.2	514	43	2	8	3
11	1955	10	10	3	25	1	90	238.8	54.4	10.8	371.7	43	2	8	4
14	1979	17	43	0	61	3	60	173	52	9.19	393	31	0	8	1
14	1979	8	14	0	23	1.5	90	184	51	10.9	464	31	0	8	2
14	1979	17	32	0	50	3	60	331	45	9.1	506	31	0	8	3
14	1979	8	9	0	18	1.5	90	172	48	10.5	462	31	0	8	4
25	1956	20	40	2	62	3	70	301.8	49.1	10	386.1	29	0	10	1
25	1956	3	2	2	7	1	100					29	0	10	2
25	1956	20	40	2	62	3	70					29	0	10	3
25	1956	3	2	2	7	1	100	243.3	47.2	14.7	607.6	29	0	10	4
27	1954	25	63	4	94	4	60	474.7	43.3	10.1	475	78	4	12	1
27	1954	13	37	4	56	1.5	90	260.5	44.9	10.7	485	78	4	12	2
27	1954	25	53	4	84	4	60	411.2	32.7	8.5	559.6	78	4	12	3
27	1954	13	24	4	43	1.5	90	217.8	45.9	9.5	446.4	78	4	12	4
28	1959	17	40	0	58	2	60	401.7	43.2	12.2	566.9	68	0	12	1
28	1959	18	21	0	40	1	90	296.6	45.8	11.3	474.5	68	0	12	2
28	1959	11	27	7	46	1.5	80	227.1	48.6	10.4	431.2	68	0	12	3
28	1959	11	4	0	16	1	100	198.3	46.8	9	376.2	68	0	12	4
38	1957	19	28	2	49	2.5	70	285.1	42.2	14.2	675.2	63	3	5	1
38	1957	5	15	2	22	1.5	90	216.7	47.6	11.6	509.7	63	3	5	2
38	1957	19	16	2	37	2.5	70	380.4	42.8	14.4	638.9	63	3	5	3
38	1957	5	5	2	12	1.5	90	187.3	44.5	10	482.6	63	3	5	4
41	1980	18	37	0	55	2	80	267.7	41.9	8.8	383.7	23	0	7	1
41	1980	1	7	0	8	1	100	183.5	47.3	8.4	311.7	23	0	7	2
41	1980	18	35	0	53	2	80	244.4	43.3	14.4	656.7	23	0	7	3
41	1980	1	5	0	6	1	100	182	51	10.6	377.2	23	0	7	4
45	1944	15	42	0	62	3	60	255	44	8.8	396	63	0	7	1
45	1944	13	21	0	39	1.5	90	336	44	10.3	459	63	0	7	2
45	1944	15	27	0	47	3	60	331	43	9.3	406	63	0	7	3
45	1944	13	12	0	30	1.5	90	241	46	10.7	458	63	0	7	4
63	1960	23	26	2	54	3	70	227.6	42.3	15.8	701.5	89	0	12	1
63	1960	13	18	2	36	1.5	90	207.4	41.8	13.4	626.79	89	0	12	2
63	1960	23	26	2	54	3	70	187.7	44.3	14.2	604.79	89	0	12	3
63	1960	13	16	2	34	1.5	90	258.89	42.9	8.19	357.7	89	0	12	4
64	1957	24	49	2	77	4	60	230.3	46.6	7.7	382.7	104		15	1
64	1957		29	2	33	2	80					104		15	2
64	1957		39	2	43	4	60					104		15	3
64	1957	12	23	2	39	2	80	194.1	43.2	7.2	326.89	104		15	4

## Appendix B

**Table B1:** Comparison of different classifiers with accuracy (ACC) and Matthews correlation coefficient (MCC) for different classifiers.

Fold#	Alglorithm	ACC	MCC
4 fold	Bayes	0.425	0.25730532
4 fold	Decision Tree	0.45	0.26164055
4 fold	Random Forest	0.625	0.50898705
4 fold	Tree Ensemble	0.65	0.53175848
4 fold	WEKA-Decision Table	0.775	0.70116894
4 fold	WEKA-Random Forest	0.45	0.27670875
40 fold	Bayes	0.525	0.38539238
40 fold	Decision Tree	0.62162	0.49525773
40 fold	Random Forest	0.675	0.56408389
40 fold	Tree Ensemble	0.725	0.63448742
40 fold	WEKA-Decision Table	0.775	0.7007412
40 fold	WEKA-Random Forest	0.55	0.38935439
5 fold	Bayes	0.525	0.36809666
5 fold	Decision Tree	0.41026	0.21908988
5 fold	Random Forest	0.6	0.46007798
5 fold	Tree Ensemble	0.7	0.60544001
5 fold	WEKA-Decision Table	0.775	0.69943346
5 fold	WEKA-Random Forest	0.6	0.46723502
6 fold	Bayes	0.53	0.33183479
6 fold	Decision Tree	0.51	0.17393888
6 fold	Random Forest	0.68	0.55837477
6 fold	Tree Ensemble	0.65	0.49267634
6 fold	WEKA-Decision Table	0.48	0.7007412
6 fold	WEKA-Random Forest	0.63	0.49387519
6 fold/SMOTE	Bayes	0.95	0.9339666
6 fold/SMOTE	Decision Tree	0.93082	0.90779989
6 fold/SMOTE	Random Forest	0.975	0.96619735
6 fold/SMOTE	Tree Ensemble	0.975	0.96675826
6 fold/SMOTE	WEKA-Decision Table	0.9125	0.88469729
6 fold/SMOTE	WEKA-Random Forest	0.95625	0.94205299

## Appendix C

**Table C1:** Comparison rough set classifiers with different fold number, accuracy (ACC), Matthews correlation coefficient (MCC).

Fold#	Dataset	Decision Class	Algorithm	ACC	MCC
6 fold	no RS	session	decomposition tree	0.53	0.347106
5 fold	no RS	session	decomposition tree	0.257	0.138529
4 fold	no RS	session	decomposition tree	0.156	0.036866
40 fold	no RS	session	decision rules	0.25	0.017689
6 fold	RS + PDQ	session	decomposition tree	0.944	0.951147
5 fold	RS + PDQ	session	decomposition tree	0.777	0.745525
4 fold	RS + PDQ	session	decomposition tree	0.783	0.789729
40 fold	RS + PDQ	session	decision rules	0.5	0.323653
6 fold	RS + PDQ	total UPDRS	decomposition tree	0.903	0.854696
5 fold	RS + PDQ	total UPDRS	decomposition tree	0.627	0.434957
4 fold	RS + PDQ	total UPDRS	decomposition tree	0.818	0.780431
40 fold	RS + PDQ	total UPDRS	decision rules	0.675	0.549481
6 fold	RS + PDQ	UPDRS II	decomposition tree	0.62	0.244933
5 fold	RS + PDQ	UPDRS II	decomposition tree	0.83	0.736768
4 fold	RS + PDQ	UPDRS II	decomposition tree	0.83	0.699704
40 fold	RS + PDQ	UPDRS II	decision rules	0.7	0.661798
6 fold	RS + PDQ	UPDRS III	decomposition tree	0.817	0.583869
5 fold	RS + PDQ	UPDRS III	decomposition tree	0.543	0.432082
4 fold	RS + PDQ	UPDRS III	decomposition tree	0.506	0.33002
40 fold	RS + PDQ	UPDRS III	decision rules	0.65	0.507846
6 fold	with RS	session	decomposition tree	0.91	0.84503
5 fold	with RS	session	decomposition tree	0.85	0.83524
4 fold	with RS	session	decomposition tree	0.838	0.724788
40 fold	with RS	session	decision rules	0.5	0.323653
